# The Treatment of Hæmoptysis

**Published:** 1904-09-17

**Authors:** 


### THE TREATMENT OF HÆMOPTYSIS.

In an article on the treatment of hæmoptysis and allied conditions, Dr. Francis Hare [1] points out that when hæmorrhage occurs in an area inaccessible to direct local treatment, relief is only to be obtained either by causing contraction of the arterioles supplying the bleeding area, or by producing vaso-dilation in other areas, with consequent fall of blood-pressure. Discussing the value of agents which cause general vaso-constriction of the arterioles, *e.g.*, ergot and adrenalin, he urges that unless the action of these is compensated for by cardiac inhibition, the blood-pressure at the site of the hæmorrhage—as, indeed, in the system generally—must be increased, with the risk of aggravation of the hæmorrhage; whilst a degree of cardiac inhibition sufficient to neutralise the rise of blood-pressure consequent on general vaso-constriction, has dangers of its own, as, for example, convulsions due to anæmia of the brain. Hence, he suggests, that the more safe and effective method of treating hæmorrhages outside the reach of local relief is to use vaso-dilating agents. These, by increasing the general arterial calibre, diminish blood-pressure and thus favour cessation of hæmorrhage and give an opportunity for the formation of blood clot in the mouths of the ruptured vessels. Putting this principle into operation he has treated several cases of hæmoptysis by the inhalation of amyl nitrite. In three of these the cause of the hæmoptysis was phthisis pulmonalis and in the fourth mitral regurgitation, and in each instance the use of the remedy was followed by immediate cessation of the hæmorrhage. The *modus operandi* is assumed to be a dilatation of the peripheral systemic arterioles causing diminished resistance to the aortic outflow and a consequent lowering of tension in the left auricle and a fall of blood-pressure in the pulmonary circulation. In short, the use af amyl nitrite in cases of hæmoptysis rests on the same physiological basis as the older practice of venesection in like circumstances, but with this advantage, that it involves no loss of blood. Treatment of hæmoptysis by morphine is no doubt often successful, but it means retention of effused blood in greater or less quantity in the lung tissue, with the risk of decomposition, septic pneumonia, high temperatures, etc.—the truth, in short, at the bottom of Niemeyer's doctrine of *phthisis ab hæmoptöe.* Dilatation of the systemic arterioles, as by the use of amyl nitrite, on the other hand, checks the hæmorrhage at the very point of its occurrence, and so prevents the escape of blood into the lung tissue. Dr. Hare has previously argued[2] that the pain in gastralgia and in migraine, as also the breathlessness of asthma, depend upon local vaso-distention consequent on widespread vaso-constriction in other areas, and he proposes that amyl nitrite or its congeners should be tried in these conditions. Another interesting suggestion is that the mechanism of menstruation is of the same order. In the immediately pre menstrual period, it is held, there is an increase in the normal systemic blood-pressure due to widespread vaso-constriction, which is met in part by vascular distention of the uterine mucosa and by the menstrual discharge. It is this pre-menstrual rise of blood-pressure which accounts for the frequency of various paroxysmal neuroses before and during the menstrual period. If this view be correct in relation to the dependence of the menstrual flow upon a general rise of the vascular tone in the cutaneous and other areas, it should follow that the use of amyl nitrite should diminish or suppress the menstrual discharge. To test this for mere purposes of experiment would hardly be legitimate, more particularly in view of the fact that such suppression may be prejudicial to the patient. But in treating a case of angina pectoris in a young woman, Dr. Hare learned that whenever amyl nitrite had been used whilst menstruation was in process the period had abruptly ceased. In principle Dr. Hare's suggestion in reference to the treatment of hæmoptysis is identical with what is often advised, namely, the use of purgatives, as calomel and magnesium sulphate, and the application of hot bottles to the feet. Here also there is an attempt to produce vaso-dilation in remote areas—the intestines and the lower limbs—with a view to reduce the blood-pressure at the site of the hæmorrhage. The same principle is illustrated in the treatment of epistaxis in elderly patients by the sustained use of the hot pediluvium, the temperature of the water being well maintained. Mr. Hutchinson[3] lays great stress upon the value of this method, and says that since he has adopted it he has never had to plug the nostrils in any case of epistaxis; he also approves the measures advised by the late Sir Andrew Clark in such cases, namely, the administration of saline purgatives and iodide of potassium. Reverting to the treatment of hæmoptysis, Dr. T. H. Green[4] some years ago issued a warning against the routine use of ergot and stated that he had lost all faith in it as a remedy for hæmoptysis. Dr. Green laid great stress on absolute quiet for the patient, the administration of purgatives, and the hypodermic use of morphine. He also referred to the use of nitrites and considered that their accelerating action on the heart rendered them of doubtful utility. This defect he had endeavoured to meet by using nitro-glycerine in combination with digitalis, the latter to neutralise the tendency to increased cardiac activity. The local application of an ice-bag to the chest Dr. Green has not found useful. Another proposal in the treatment of hæmoptysis is that the chest should be strapped on the affected side. This was advanced by Neidner.[5] He reports five cases in which this manœuvre was successful. The chest was strapped with 12 lengths of rubber plaster, each $\frac{3}{4}$ inch wide and 2 feet long. In only one instance was the success incomplete, and in this the patient insisted on removing the strapping.

[1] Lancet, August 20, 1904. [2] Australian Med. Gazette, July-Oct., 1903. [3] Archives of Surgery, Vol. IV., p. 180. [4] Clin. Jour., Vol. II. [5] Brit. Med. Jour. (Epitome), Nov. 1, 1902.

